# Salivary gland proteome analysis of developing adult female *Haemaphysalis longicornis* ticks: molecular motor and TCA cycle-related proteins play an important role throughout development

**DOI:** 10.1186/s13071-019-3864-2

**Published:** 2019-12-30

**Authors:** Shuguang Ren, Baowen Zhang, Xiaomin Xue, Xiaoshuang Wang, Huaqu Zhao, Xiaoli Zhang, Minjing Wang, Qi Xiao, Hui Wang, Jingze Liu

**Affiliations:** 1 Hebei Key Laboratory of Animal Physiology, Biochemistry and Molecular Biology, College of Life Sciences, Hebei Normal University, Shijiazhuang, 050024 China; 2 The Fourth Hospital of Hebei Medical University, Shijiazhuang, 050011 China

**Keywords:** Tick, Salivary gland, Molecular motor, iTRAQ, RNA interference (RNAi)

## Abstract

**Background:**

Ticks are notorious blood-feeding arthropods that can spread a variety of deadly diseases. The salivary gland is an important organ for ticks to feed on blood, and this organ begins to develop rapidly when ixodid ticks suck blood. When these ticks reach a critical weight, the salivary glands stop developing and begin to degenerate. The expression levels of a large number of proteins during the development and degeneration of salivary glands change, which regulate the biological functions of the salivary glands. Furthermore, to the best of our knowledge, there are only a few reports on the role of molecular motor and TCA cycle-related proteins in the salivary glands of ticks.

**Results:**

We used iTRAQ quantitative proteomics to study the dynamic changes in salivary gland proteins in female *Haemaphysalis longicornis* at four feeding stages: unfed, partially fed, semi-engorged and engorged. Using bioinformatics methods to analyze the dynamic changes of a large number of proteins, we found that molecular motor and TCA cycle-related proteins play an important role in the physiological changes of the salivary glands. The results of RNAi experiments showed that when dynein, kinesin, isocitrate dehydrogenase and citrate synthase were knocked down independently, the weight of the engorged female ticks decreased by 63.5%, 54.9%, 42.6% and 48.6%, respectively, and oviposition amounts decreased by 83.1%, 76.0%, 50.8%, and 55.9%, respectively, and the size of type III acini of females salivary glands decreased by 35.6%, 33.3%, 28.9%, and 20.0%, respectively.

**Conclusions:**

The results showed that the expression of different types of proteins change in different characteristics in salivary glands during the unfed to engorged process of female ticks. Corresponding expression changes of these proteins at different developmental stages of female ticks are very important to ensure the orderly development of the organ. By analyzing these changes, some proteins, such as molecular motor and TCA cycle-related proteins, were screened and RNAi carried out. When these mRNAs were knocked down, the female ticks cannot develop normally. The research results provide a new protein target for the control of ticks and tick-borne diseases.

## Background

Ticks are harmful external parasitic arthropods that transmit a variety of pathogenic microorganisms to their hosts while sucking blood. These pathogens can lead to the development of many lethal diseases in hosts, such as Lyme disease, human anaplasmosis, babesiosis, and tick-borne encephalitis [1]. To adapt to a blood-feeding lifestyle, ticks have evolved special body structures and blood-feeding organs with unique functions. Among them, the salivary glands are important for successful blood-feeding, because they secrete saliva that includes large amounts of powerful pharmacologically active proteins, which can inhibit host blood coagulation [2, 3], immune response [4] and vascular repair [5]. Salivary glands are also the main organs involved in tick-borne diseases, because saliva contains a large number of pathogens [6, 7].

The salivary glands of different female ixodidae tick species exhibit similar developmental characteristics. The salivary glands of female ticks are composed of different types of acini (I, II, III) with ducts connected to each other [8]. Salivary glands have the ability to regulate water balance during the unfed stage. When a tick finds a host and pre-attachment occurs, some genes are upregulated in the salivary glands [9–11]. The development and functional adjustment of the salivary glands are more related to blood-feeding. When the tick starts to suck blood, the salivary glands begin to develop rapidly, and expressions of a large number of genes are upregulated rapidly. However, the salivary glands begin to degenerate when the female tick reaches a critical weight [12–15]. Salivary gland degeneration is caused by the ecdysteroid hormone [16], which is also regulated by many proteins [17].

The coordination of various proteins plays a decisive role in the development and degeneration of salivary glands. Analyzing global changes in salivary gland proteins is critical to develop more effective molecular control methods. Quantitative proteomics was used to rapidly monitor global proteins in the development of salivary glands. Some studies on the proteome of tick salivary glands of *Hyalomma* and *Amblyomma* genus have been reported [18–20], but there are no studies on global proteins in the feeding cycle of female adult of tick salivary glands. In the present study, we focused on the expression changes of global proteins in salivary glands during the feeding cycle of female adult *Haemaphysalis longicornis*, providing deeper insights into the molecular mechanisms of tick salivary gland development and degeneration. *Haemaphysalis longicornis* is widely distributed in East Asia and Oceania [21] and is very harmful to animal husbandry and human health. This species is a major vector of multiple pathogens, such as *Babesia*, *Rickettsia*, *Theileria*, and severe fever with thrombocytopenia syndrome virus (SFTSV) [22, 23].

The widely used isobaric tags for relative and absolute quantification (iTRAQ) quantitative proteomics method has many advantages and can be used for large-scale and accurate quantitative studies of multicomponent samples. In the present study, we determined the protein expression kinetics of the salivary glands of adult *H. longicornis* females during the four feeding stages: unfed, partially fed, semi-engorged and engorged. In total, we identified 5831 salivary gland proteins in the four feeding stages. The expression of many proteins related to energy production and material transport was significantly increased in the salivary glands of female ticks after they started to suck blood. The increased expression of these proteins provides sufficient energy and materials for the rapid development of the salivary glands. These proteins were then individually knocked down with RNAi.

The results showed that after knockdown of the proteins dynein, kinesin, isocitrate dehydrogenase, and citrate synthase, female ticks were unable to suck blood normally. In particular, 52.5% and 57.5% of ticks died after silencing of dynein and kinesin. There are few reports on these four proteins regulating the function of tick salivary glands. Dynein and kinesin, which are molecular motor proteins, play an important role in mitosis, meiosis and transport of cellular cargo [24–26]. In addition, isocitrate dehydrogenase and citrate synthase are indispensable proteins of the tricarboxylic acid (TCA) cycle [27]. These proteins provide sufficient energy during blood-feeding to enable rapid expansion of the tick body.

## Methods

### Tick breeding and protein extraction

*Haemaphysalis longicornis* were captured from Xiaowutai Mountain National Nature Reserve of China. Ticks were allowed to feed on the ears of New Zealand white rabbits by using earmuffs made of white cloth. Rabbits were kept in constant temperature culture room (25 ± 1 °C). When not sucking blood, the ticks were cultured in artificial climate incubators at 25 ± 1 °C and 75% relative humidity. Female adult ticks in four feeding stages, unfed (approximately 1.8 ± 0.1 mg, 0.32 ± 0.02 cm), partially fed (approximately 2.5 days post-feeding, 11.9 ± 0.4 mg, 0.48 ± 0.02 cm), mated semi-engorged (approximately 4 days post-feeding, 40.1 ± 0.8 mg, 0.67 ± 0.02 cm) and engorged (approximately 5.5 days post-feeding, 249.2 ± 6.8 mg, 0.99 ± 0.02 cm), were collected for this study. *Haemaphysalis longicornis* is a rather small tick, therefore, in order to obtain enough proteins for this experiment, approximately 500 adult female ticks were needed per group for experiments (approximately 6000 ticks in total). Approximately 50 New Zealand white rabbits (*Oryctolagus cuniculus*) were used to feed the ticks and the salivary glands were dissected from the ticks at each feeding stage. After being washed with PBS (0.01 M), the salivary glands were deposited immediately in PBS buffer containing cOmplete™ protease inhibitor cocktail (Roche, Mannheim, Germany) and quickly frozen at − 80 °C. When enough salivary glands were collected, protein extraction was performed as previously described [28]. The salivary glands were fully ground in a glass homogenizer then transferred into a 50 ml centrifuge tube and centrifuged for 20 min (4 °C, 12,000×*g*). Then, supernatant was collected and an equal volume of Tris-saturated phenol (pH 7.8) was added to the supernatant. The sample was mixed thoroughly and centrifuged for 20 min (4 °C, 12,000×*g*). Then, an equal volume of 50 mM Tris-HCl (pH 8.0) was added and the solution mixed thoroughly, followed by centrifugation for 20 min (4 °C, 12,000×*g*). Then 0.1 M ammonium acetate in methanol was added to the solution after the supernatant was removed and stored at − 20 °C overnight. Then the solution was centrifuged for 20 min (4 °C, 12,000×*g*), and the supernatant removed. The protein pellets were washed again with methanol and were lyophilized and stored at −80 °C.

### Protein digestion and iTRAQ labeling

**Fig. 1:**
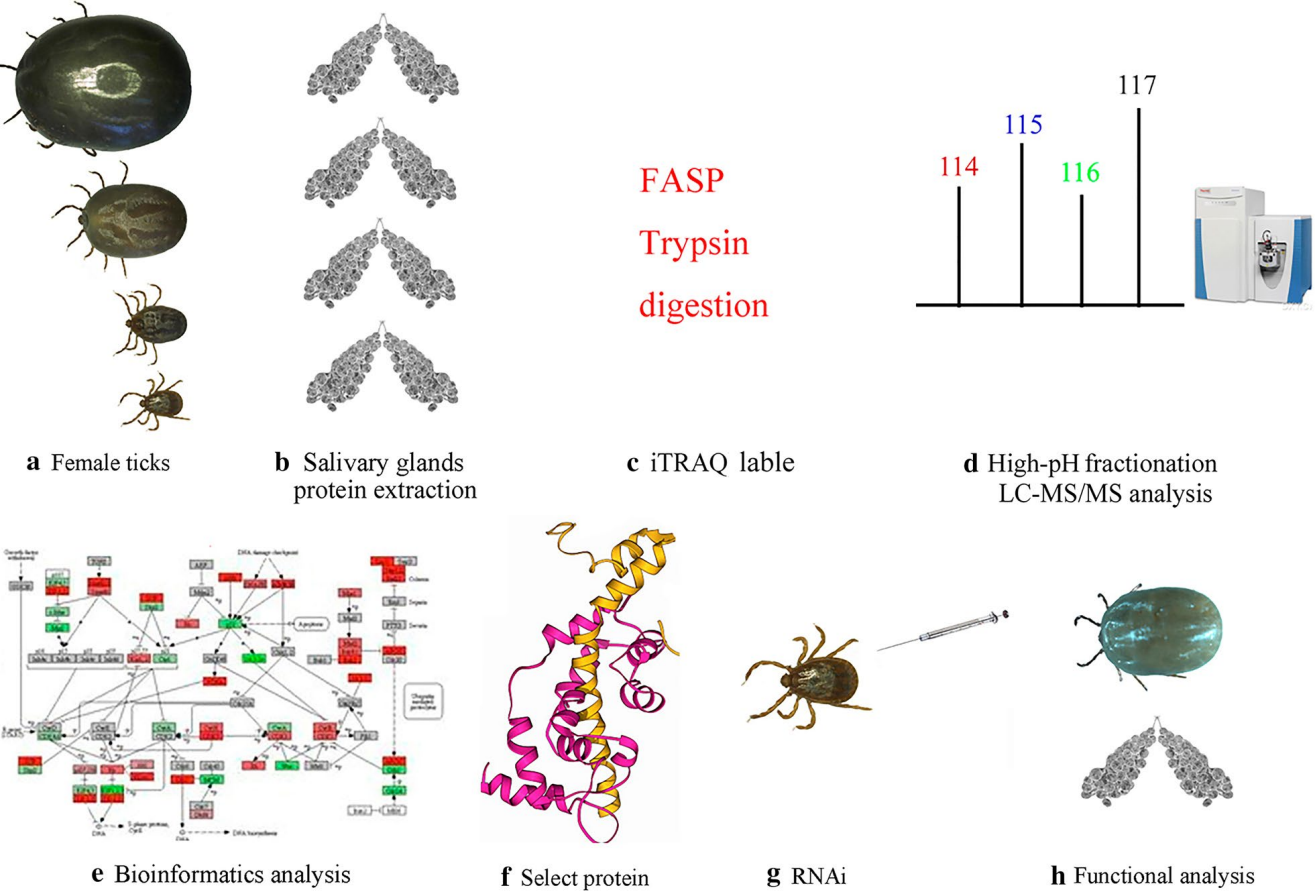
Workflow for quantitative proteomics analysis of changes in protein expression in the salivary glands of female *H. longicornis* during the blood-feeding process

Protein digestion was performed as previously described [28]. Salivary gland protein samples (200 μg) from each blood-feeding stage were reduced with 10 mM dithiothreitol and then alkylated with 20 mM iodoacetamide. Then, the samples were processed using a filter-aided sample preparation (FASP) protocol [29]. Enzymatic digestion was performed with sequencing-grade modified trypsin (1:2 w/w, Promega, Madison, USA) at 37 °C (strictly controlled) for 12 h. After digestion, the peptides were eluted (centrifugation at 12,000×*g* for 20 min) from the ultrafiltration membrane with iTRAQ dissolution buffer (AB SCIEX, Redwood, USA). The concentrations of the trypsin-digested peptides were detected with a NanoDrop spectrophotometer (Thermo Fisher Scientific, Waltham, USA) and an LC-MS system (Thermo Fisher Scientific) so that the components could be adjusted to equal concentrations. Enzyme efficiency was also monitored by LC-MS. Figure 1 shows the experimental workflow using iTRAQ. Each sample was labeled with iTRAQ (AB SCIEX) 4-plex reagents (114–117) according to the manufacturer’s instructions, respectively. After deionized water was added to stop the reaction, the four labeled samples were mixed together for further analysis.

### High-pH reversed-phase (RP) fractionation

The iTRAQ-labeled peptide mix samples were separated by high-pH (pH = 10) C18 (Agela; 5 μm particle size, 100 Å pore size, 0.46 × 25 cm) reversed-phase high-performance liquid chromatography (C18 RP-HPLC). The peptides were eluted at a gradient flow of 1 ml/min, and the concentration of solvent B (solvent A: H_2_O containing 5 mM ammonium formate, pH 10.0; solvent B: acetonitrile (ACN) containing 5 mM ammonium formate, pH 10.0) in the elution solvent was raised to 60% (v/v) over 70 min. A tube of elution components was collected every minute and randomly combined. As a result, 30 eluted fractions were ultimately obtained for further LC-MS analysis.

### LC-MS analysis

Peptide mixtures in each eluted fraction were analyzed with a Q Exactive HF (Thermo Fisher Scientific) mass spectrometer coupled online to a nanoACQUITY UPLC M-Class system (waters, Milford, USA) as previously described [28]. Each sample was desalted with a C18 RP trap column (5 μm particle size, 100 Å pore size, 180 μm ID × 20 mm length; Waters, Milford, USA) and then separated with a C18 RP analytical column (1.8 μm particle size, 100 μm ID × 150 mm length; Waters) at a flow rate of 300 nl/min using a linear gradient (0–40%) of solvent B (solvent A: 99.9% H_2_O + 0.1% formic acid; solvent B: 99.9% ACN + 0.1% formic acid) over 75 min. LC-MS data were acquired in the data-dependent acquisition mode. All mass spectrometry parameters were set as previously described [28]. Three biological replicates were performed for iTRAQ analysis.

### Protein identification and iTRAQ quantification

Thirty files (raw data) identified by mass spectrometry (MS) were combined into one MS/MS dataset and then analyzed using the SEQUEST algorithm embedded in Proteome Discoverer (version 2.1) software (Thermo Fisher Scientific). The *H*. *longicornis* proteins database derived from transcriptome sequencing (GenBank: GHLT00000000). Ticks were raised in laboratory conditions since they were collected from Xiaowutai. The ticks used in this study have been bred in the laboratory for more than 3 generations and fed on the ears of New Zealand white rabbits. In order to eliminate any contamination, host *O. cuniculus* and human sequences were used as a contaminated database for proteomic searching. Search parameters were applied as previously described [28]: (i) a precursor mass tolerance of 10 ppm was used; (ii) a fragment mass tolerance of 0.5 Da was used; (iii) trypsin was set as the enzyme, and 2 missed cleavages were allowed; (iv) methionine oxidation was set as the variable modification; (v) the iTRAQ 4-plex reagents (N terminus and lysine residues, 144.102 Da) were defined, and carbamidomethylation of cysteine residues was set as the fixed modification; (vi) high-energy collision-induced dissociation (HCD) was chosen as the activation type; (vii) the “total peptide amount” tab was checked to normalize the protein levels by the protein ratio median; and (viii) a decoy database search was simultaneously performed to estimate the false discovery rate (FDR). The q-value was used as the criterion to evaluate FDR, and the target FDR was set to 0.01. Only quantified proteins with at least two unique peptides and high confidence (FDR < 0.01) were considered for further analysis. Protein quantification was based on the normalized spectrum abundance factor (NSAF). Proteins with expression changes greater than 1.5-fold were considered to be upregulated or downregulated.

### Bioinformatics analysis

To further reveal the functions of the proteins, a number of bioinformatics analyses were carried out. The GProX platform was used to cluster the salivary gland proteins with similar expression patterns in the 4 tick feeding stages [30]. The number of clusters was set to 4, and a fixed regulation threshold (upper limit of 0.58 and lower limit of − 0.58, corresponding to the original ratios of approximately 1.5 and 0.67) was used [31, 32]. The minimal membership for the plot was set as 0.5. Other parameters were set to default values. PANTHER classification (http://www.pantherdb.org/) was used to carry out a Gene Ontology (GO) functional annotation. The pathways of the differentially expressed proteins (*P*-values < 0.05) were carried out using the Kyoto Encyclopedia of Genes and Genomes (KEGG) database (http://www.kegg.jp/kegg/) and KOBAS 3 software (http://kobas.cbi.pku.edu.cn/).

### RNA interference

T7 RiboMAX^TM^ Express RNAi System (Promega) was used to synthesize double-stranded RNA (dsRNA) according to the manufacturer’s instructions. Specific *H. longicornis* nucleotide sequence targeting positions 13456–13965, 104–515, 235–763, and 649–1291 of the dynein heavy chain (GenBank: GHLT01015731), the kinesin heavy chain (GenBank: GHLT01011136), isocitrate dehydrogenase (GenBank: GHLT01012103), and citrate synthase (GenBank: GHLT01017326), respectively, were selected for dsRNA synthesis. After the target mRNA sequences were cloned and sequenced, the correct target cDNA sequences for RNAi were used for dsRNA synthesis. The primers used to synthesize dsRNA included the T7 promoter sequence (underlined) were as follows: 5′-TAA TAC GAC TCA CTA TAG GCA GTG GGT GAC GGA TTT CTC-3′ and 5′-TAA TAC GAC TCA CTA TAG GGG AAC ACA GCA GAG CGA CAC-3′ (dynein heavy chain), 5′-TAA TAC GAC TCA CTA TAG GGC AAG TTC ATC GTC AAG TTC-3′ and 5′-TAA TAC GAC TCA CTA TAG GAC TCT GTT CTT GTC CTC GTG-3′ (kinesin heavy chain), 5′-TAA TAC GAC TCA CTA TAG GAT CCC GCA GAA GGC TAT T-3′ and 5′-TAA TAC GAC TCA CTA TAG GCG TAC AAG TTG GGC ATC AC-3′ (isocitrate dehydrogenase), 5′-TAA TAC GAC TCA CTA TAG GGC CAC CAT CTA CCG CAA CC-3′ and 5′-TAA TAC GAC TCA CTA TAG GGG GAC ACT CCA AAC AGC ACC-3′ (citrate synthase). Green fluorescent protein (GFP, GenBank: KX247384.1) dsRNA was synthesized as the control [33]. Twenty unfed female *H. longicornis* were injected with 0.5–1 µl (4 µg/µl) dsRNA using a Hamilton syringe (33-gauge needle) in the lower right quadrant of the body [34]. After injection, the ticks were cultured in a humid incubator for 24 h to recover, then were allowed to feed on the ears of New Zealand white rabbits using cloth earmuffs. The degree of mRNA knockdown was confirmed by analysis of mRNA expression levels using RT-qPCR. A digital microscope (DVM6; Leica, Oskar, Germany) was used to observe morphological changes of the salivary gland acini. Ten RNAi ticks at each feeding stage were used to observe the shape and size of salivary glands. Other physiological changes of female ticks after RNAi were measured: (i) tick mortality rate; (ii) time required for engorgement (starting with biting the host) and the weight of the engorged tick; (iii) number of eggs; and (vi) egg hatching rate.

### RT-qPCR quantitative analysis

In order to observe the expression of transcription levels of these four proteins during the four feeding stages: unfed, partially fed, semi-engorged, and engorged. We performed RT-qPCR quantitative analysis of mRNA from different periods. In addition, RT-qPCR analysis was performed at 48 h after the unfed ticks were injected with dsRNA. Total RNA of salivary glands of 10 ticks were extracted using TRIzol Reagent (Invitrogen, Carlsbad, USA). After treatment with DNase, the cDNA was synthesized using the PrimeScript™ II 1st Strand cDNA Synthesis Kit according to the manufacturer’s protocol (Takara, Tokyo, Japan). The primers used for RT-qPCR were as follows: 5′-CTC CGG GTT GAA CAG GC-3′ and 5′-CAG TGG GTG ACG GAT TTC T-3’ (dynein heavy chain), 5′-GTG CTC ATG GGC TAC AA-3′ and 5′-GGG TAT GAT GCC TTG ACT-3′ (kinesin heavy chain), 5′-GCA TCC AGG AAC ATC GC-3′ and 5′-GCC GTC GGA CAT TCT CAT-3′ (isocitrate dehydrogenase), 5′-CAC CAT CTA CCG CAA CCT G-3′ and 5′-GGT CGT CGT ATC CGA GCA T-3′ (citrate synthase). Actin was used as an internal reference gene. The 2^−ΔΔCq^ equation [35] was used to calculate the gene expression silencing rate.

### Statistical analysis

The coefficients of variation (CV) were used to calculate the degree of data dispersion between repeated experiments (CV = SD/Mean × 100%), proteins abundance ratios with CV < 20% were selected for further analysis. *R*^2^ was used to analyze correlations between repeated experiments. If > 0.9, it means that the data we obtained is reproducible and accurate. The student’s *t*-test (Statistica 6.0) was used to analyze the quantitative results of RT-qPCR. If *P*-values < 0.05, it was considered that there was a significant difference in mRNA expression levels between different groups.

## Results

### Protein identification and quantitation

**Fig. 2:**
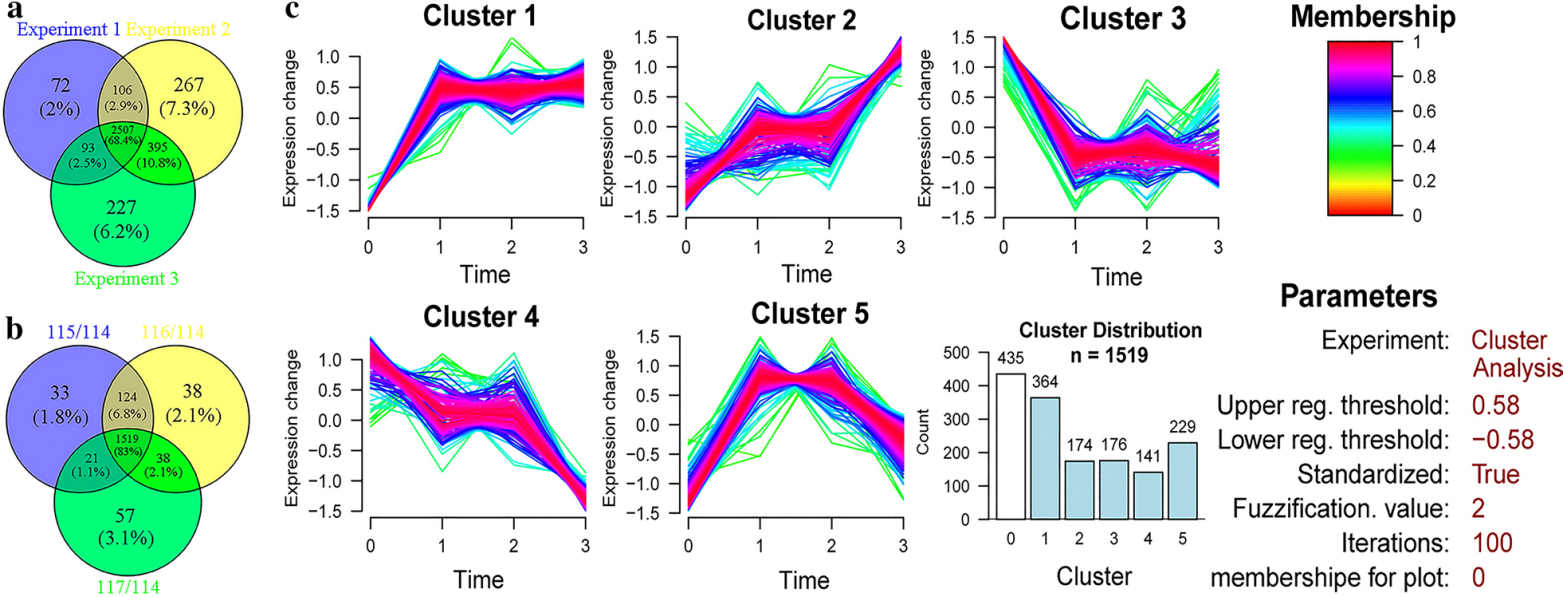
Statistics and cluster analysis for the identified proteins and their expression levels in the salivary glands of female *H. longicornis*. **a** Venn diagram showing the number of proteins (with CV < 20%) identified in the three experiments. **b** Venn diagram showing the number of proteins with quantitative information. **c** Cluster analysis according to trends in protein expression in the salivary glands of female ticks

Figure 1 shows the experimental workflow. After trypsin digestion, mass spectrometry identification and data analysis, the proteins were identified, and quantitative results were obtained (Additional file 1: Table S1, Additional file 2: Table S2, Additional file 3: Table S3, Additional file 4: Table S4). The first, second and third biological replicates identified 5059 (experiment 1), 5526 (experiment 2) and 5584 (experiment 3) proteins, respectively. Only quantified proteins with at least 2 unique peptides and high confidence (FDR < 1%) were considered for further analysis. The Venn diagram shows the overlap of proteins from three biological replicates, a total of 3667 high-confidence unique proteins were in all three experiments (Fig. 2a); 2507 high-confidence proteins were shared among all three experiments (Fig. 2a), accounting for 68.4% of the total proteins. The coefficients of variation (CV) for the partially fed:unfed protein abundance ratio (115:114), the mated semi-engorged:unfed ratio (116:114), and the engorged:unfed ratio (117:114) were calculated. Proteins whose abundance ratios with CV < 20% were selected for further analysis (Additional file 4: Table S4). log2 correlation coefficients for protein abundance ratios from the different experimental replicates were calculated. The results showed that the relative changes in protein abundance were correlated (*R*^2^ > 0.9) between different experiment replicates (Additional file 5: Figure S1).

### Cluster analysis of differentially expressed proteins

After eliminating the null values in the quantitative results, we selected proteins with CV < 20% from among the 2507 identified proteins shared among all three experiments for further analysis. The construction of clusters was based on all the quantified proteins from the 115/114, 116/114 and 117/114 comparisons (Fig. 2b). We divided these proteins into 5 clusters according to the trends in the protein expression changes in the 4 different feeding stages (Fig. 2c, Additional file 6: Table S5). Proteins with values higher than 1.5 or lower than 0.67 were considered to be upregulated or downregulated, which corresponded to log 2 values of 0.58 and − 0.58, respectively.

Proteins with similar expression patterns were grouped together. Overall, 435 proteins exhibited no significant changes in expression (Cluster 0). The expression patterns of the upregulated and downregulated proteins were generally divided into five clusters by GProX clustering analysis: Cluster 1 contained 364 proteins; Cluster 2 contained 174 proteins; Cluster 3 contained 176 proteins; Cluster 4 contained 141 proteins; and Cluster 5 contained 229 proteins. There were many different types of proteins in each cluster; for example, the overall trend of protein expression in Cluster 1 from the unfed stage to engorgement was upregulation. Then, the protein expression remained largely unchanged: dynein heavy chain (115/114 = 1.8; 116/114 = 1.8; 117/114 = 1.9); kinesin heavy chain (115/114 = 1.6; 116/114 = 1.6; 117/114 = 1.4); isocitrate dehydrogenase (115/114 = 1.9; 116/114 = 1.8; 117/114 = 1.9); citrate synthase (115/114 = 1.7; 116/114 = 1.5; 117/114 = 1.8); angiotensin-converting enzyme (115/114 = 2.3; 116/114 = 2.5; 117/114 = 3.3). The overall trend of protein expression in Cluster 2 from the unfed stage to engorgement was continuous upregulation. The proteins in Cluster 2 were mainly enzymes, such as enolase (115/114 = 2.1; 116/114 = 2.0; 117/114 = 3.2). In addition, Cluster 2 also contained certain structural proteins, such as alpha tubulin (115/114 = 3.0; 116/114 = 3.2; 117/114 = 6.0). The overall trend of protein expression in Cluster 4 from the unfed stage to engorgement was continuous downregulation. The proteins in Cluster 4 included some phosphatases, such as deoxyuridine 5′-triphosphate nucleotidohydrolase (115/114 = 0.9; 116/114 = 0.8; 117/114 = 0.5) and phosphatidylinositol-4-phosphate 5-kinase (115/114 = 0.8; 116/114 = 0.8; 117/114 = 0.6). In addition, Cluster 4 also contained certain binding proteins, such as polyadenylate-binding protein-interacting protein (115/114 = 0.9; 116/114 = 0.7; 117/114 = 0.6).

### GO function annotation

**Fig. 3:**
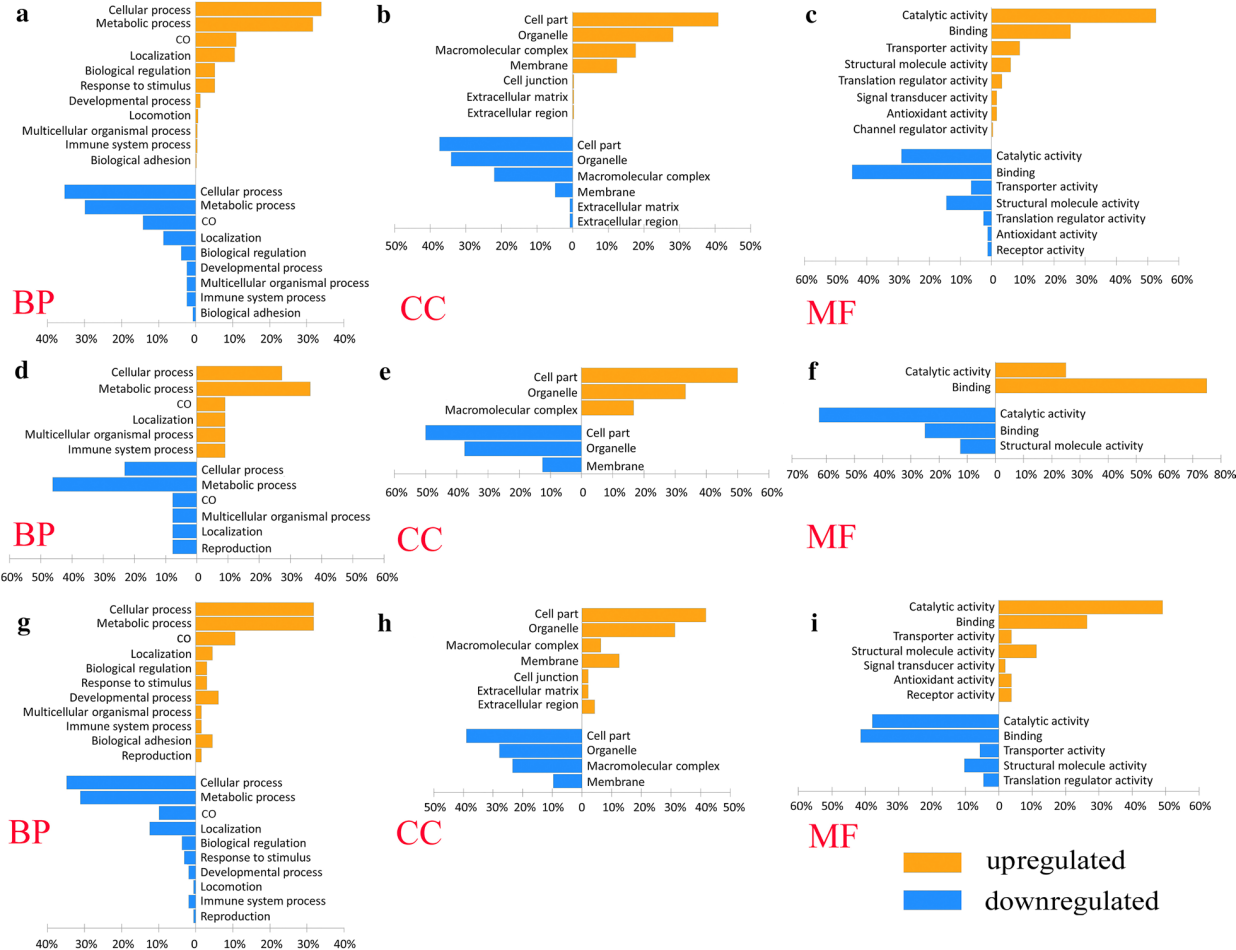
GO functional annotations for all the differentially expressed proteins. **a**–**c** GO annotations of differentially expressed proteins in the salivary glands of partially fed ticks compared with unfed ticks (115:114). **d**–**f** GO annotations of differentially expressed proteins in the salivary glands of mated semi-engorged ticks compared with partially fed ticks (116:115). **g**–**i** GO annotations of differentially expressed proteins in the salivary glands of engorged ticks compared with mated semi-engorged ticks (117:116). *Abbreviations*: BP, biological process; CC, cellular component; MF, molecular function; CO, cellular component organization or biogenesis

As shown in Fig. 3, the GO functions were annotated for all the differentially expressed proteins in the following comparisons: partially fed:unfed (115:114); mated semi-engorged:partially fed (116:115); and engorged:mated semi-engorged (117:116). The proteins were all grouped into three major functional groups: biological processes; cellular components; and molecular functions. Further enrichment analysis was conducted in these three categories (Fig. 3), the results showed that for the proteins upregulated and downregulated in partially fed ticks compared with unfed ticks, a total of 11 terms (means different kinds of biological functions) and 9 terms were enriched in the biological process category, respectively (Fig. 3a), 7 terms and 6 terms were enriched in the cellular component category, respectively (Fig. 3b), and 8 terms and 7 terms were enriched in the molecular function category, respectively (Fig. 3c). Upregulated and downregulated proteins in mated semi-engorged ticks compared with partially fed ticks show that a total of 6 terms and 6 terms were enriched in the biological process category, respectively (Fig. 3d), 3 terms and 3 terms were enriched in the cellular component category, respectively (Fig. 3e), and 2 terms and 3 terms were enriched in the molecular function category, respectively (Fig. 3f). Upregulated and downregulated proteins in engorged ticks compared with mated semi-engorged ticks show that a total of 11 terms and 10 terms were enriched in the biological process category, respectively (Fig. 3g), 7 terms and 4 terms were enriched in the cellular component category, respectively (Fig. 3h), and 7 terms and 5 terms were enriched in the molecular function category, respectively (Fig. 3i). GO annotation was also performed for the differentially expressed proteins in Clusters 1–5 (Additional file 7: Figure S2).

### KEGG pathway analysis

**Fig. 4:**
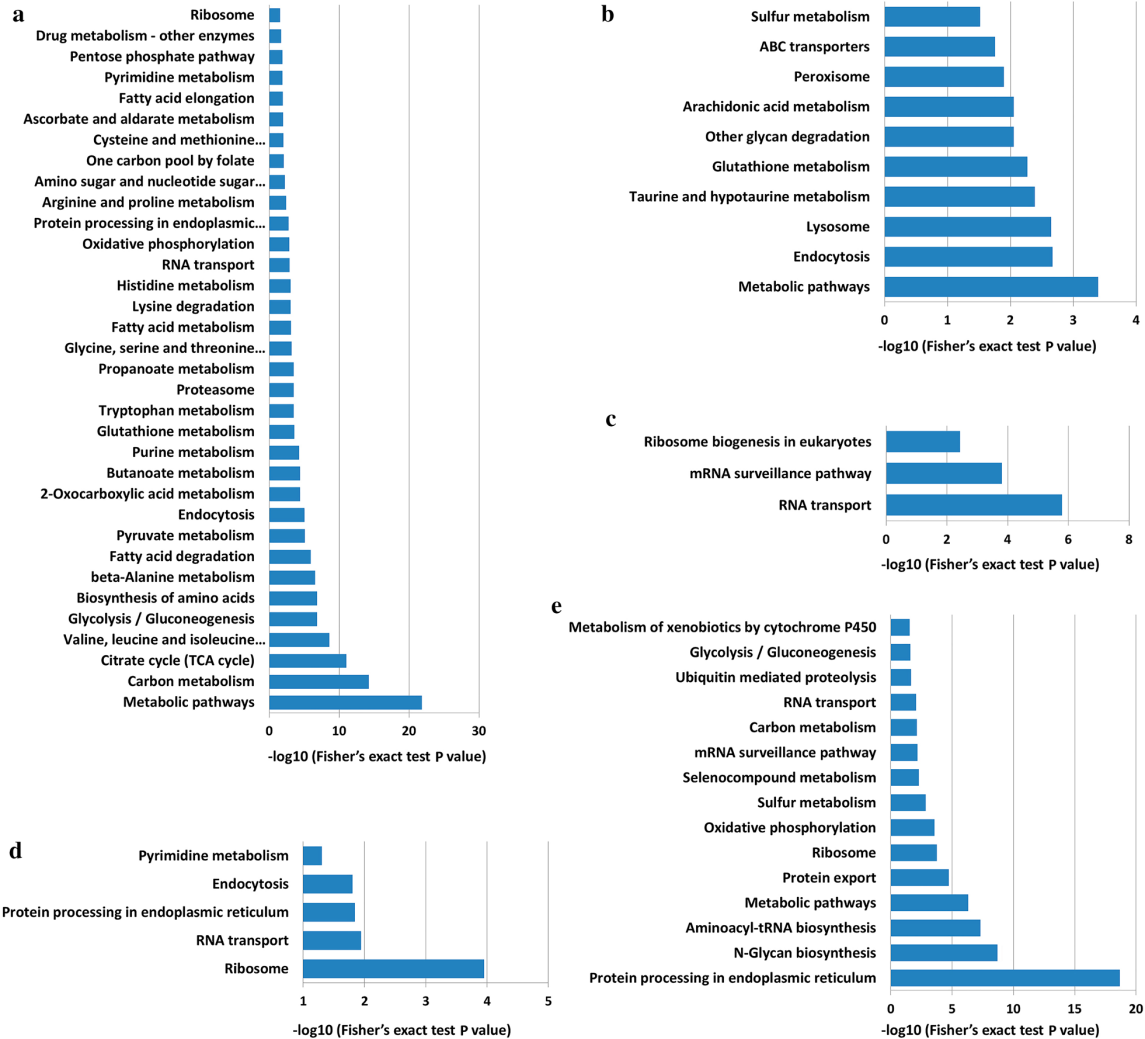
KEGG pathway enrichment analysis of the differentially expressed proteins in 5 different Clusters. Terms with a *P*-value < 0.05 were used to draw the column diagrams. **a**–**e** KEGG pathway enrichment for the proteins in Cluster 1 to Cluster 5

The differentially expressed proteins in the 5 different Clusters were subjected to KEGG pathway enrichment analysis. In Clusters 1–4, and 5, 66, 30, 22, 15, and 35 terms were mapped to KEGG pathways, respectively (Additional file 8: Table S6, Additional file 9: Table S7, Additional file 10: Table S8, Additional file 11: Table S9, Additional file 12: Table S10). Terms with *P*-values < 0.05 were used to draw column diagrams. After screening, the number of terms was reduced to 34, 10, 3, 5, and 15 for Clusters 1–5, respectively (Fig. 4). The “RNA transport” term was distributed in all four Clusters except Cluster 2; the term “endocytosis” was distributed in Clusters 1, 2, and 4; the term “metabolic pathways” was distributed in Clusters 1, 2, and 5; and the terms “ribosome” and “protein processing in endoplasmic reticulum” were distributed in Clusters 1, 4 and 5.

### Phenotypes associated with RNAi

**Fig. 5:**
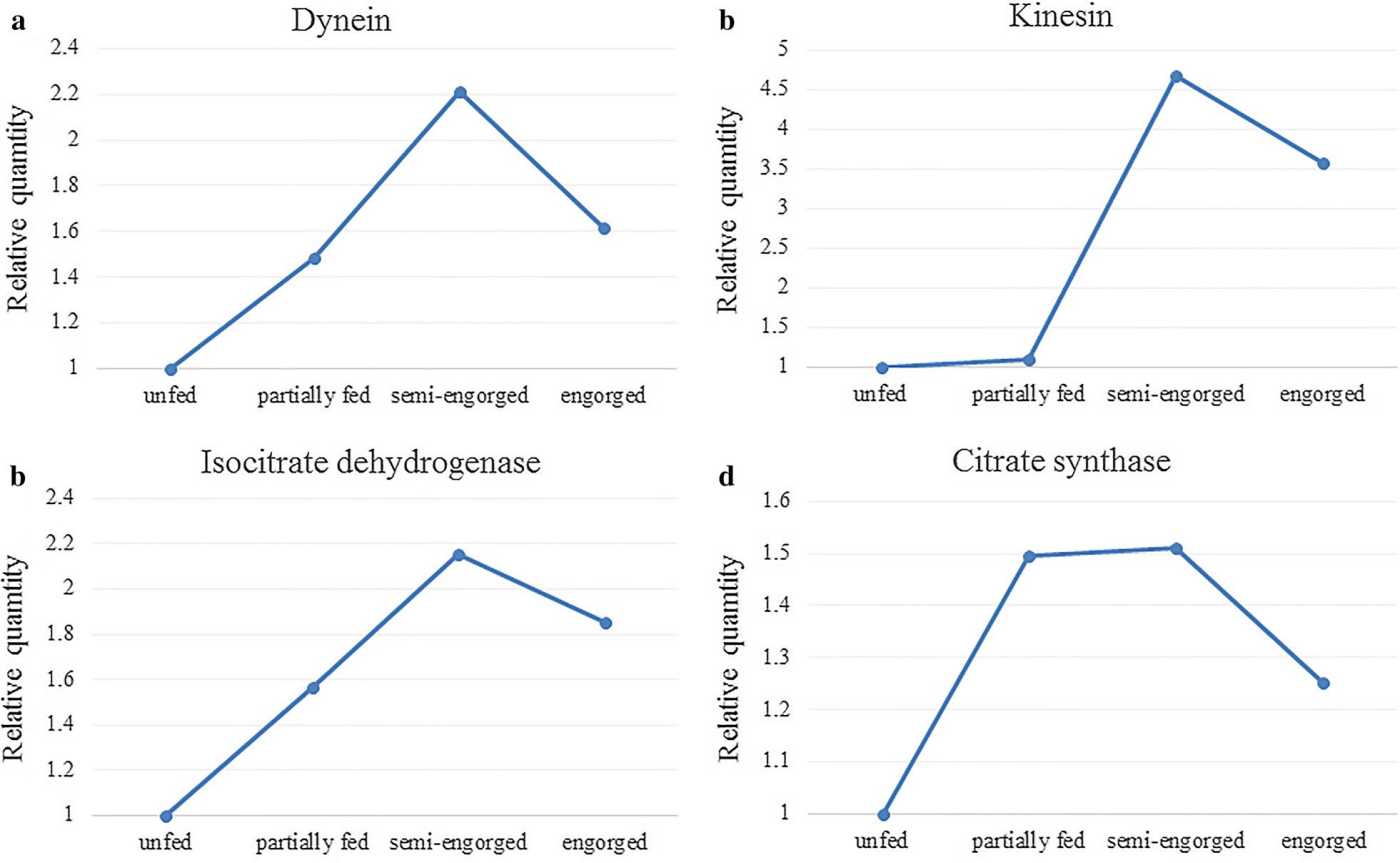
RT-qPCR analyzed the mRNA expression levels of dynein, kinesin, isocitrate dehydrogenase, and citrate synthase during the four feeding stages of salivary gland development

**Fig. 6:**
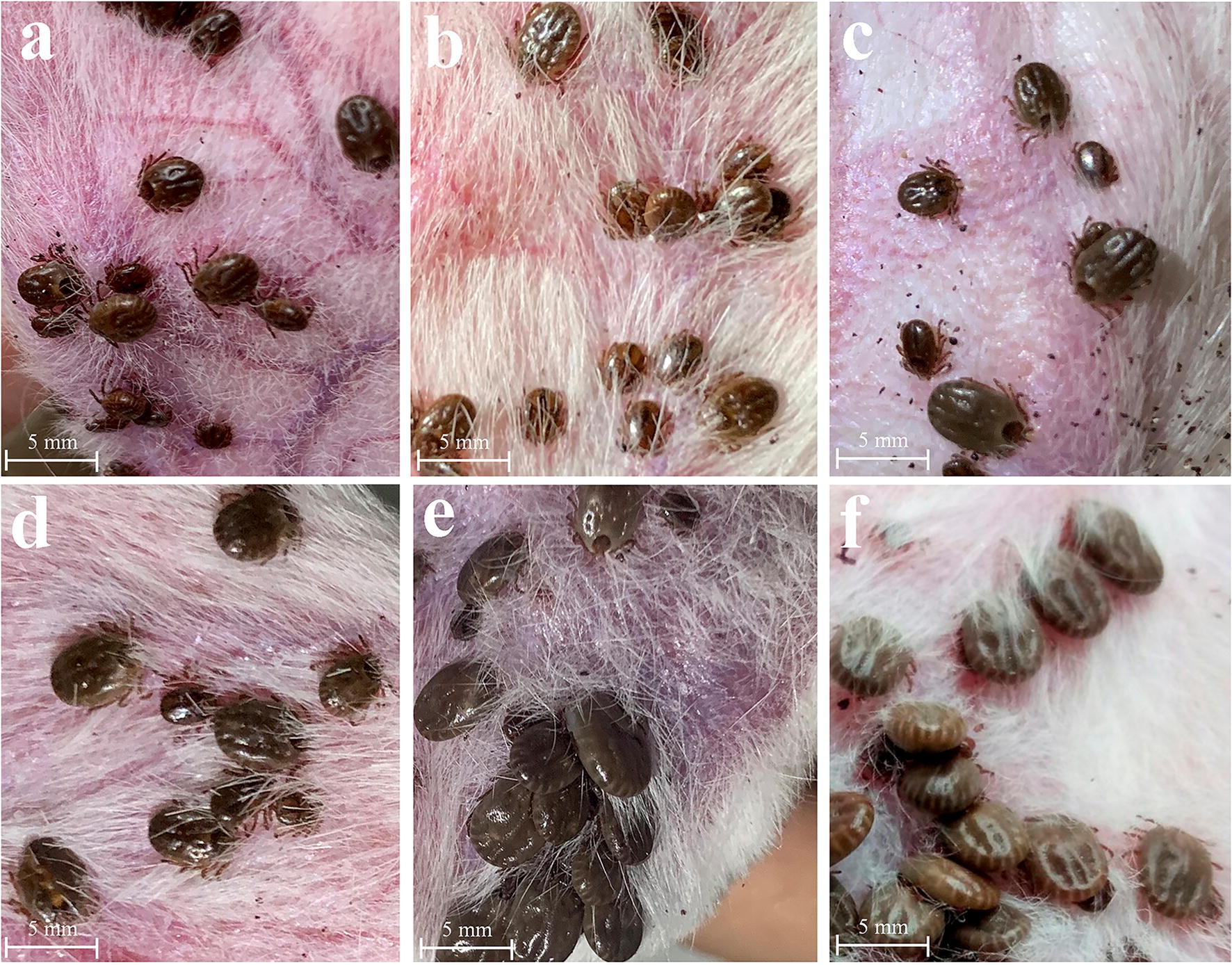
Phenotype associated with dynein, kinesin, isocitrate dehydrogenase and citrate synthase mRNA subjected to RNAi in female ticks *via* injection with the corresponding dsRNA. **a** Dynein dsRNA injection. **b** Kinesin dsRNA injection. **c** Isocitrate dehydrogenase dsRNA injection. **d** Citrate synthase dsRNA injection. **e** GFP dsRNA injection, control. **f** No injection, control. *Scale-bars*: 5 mm

**Fig. 7:**
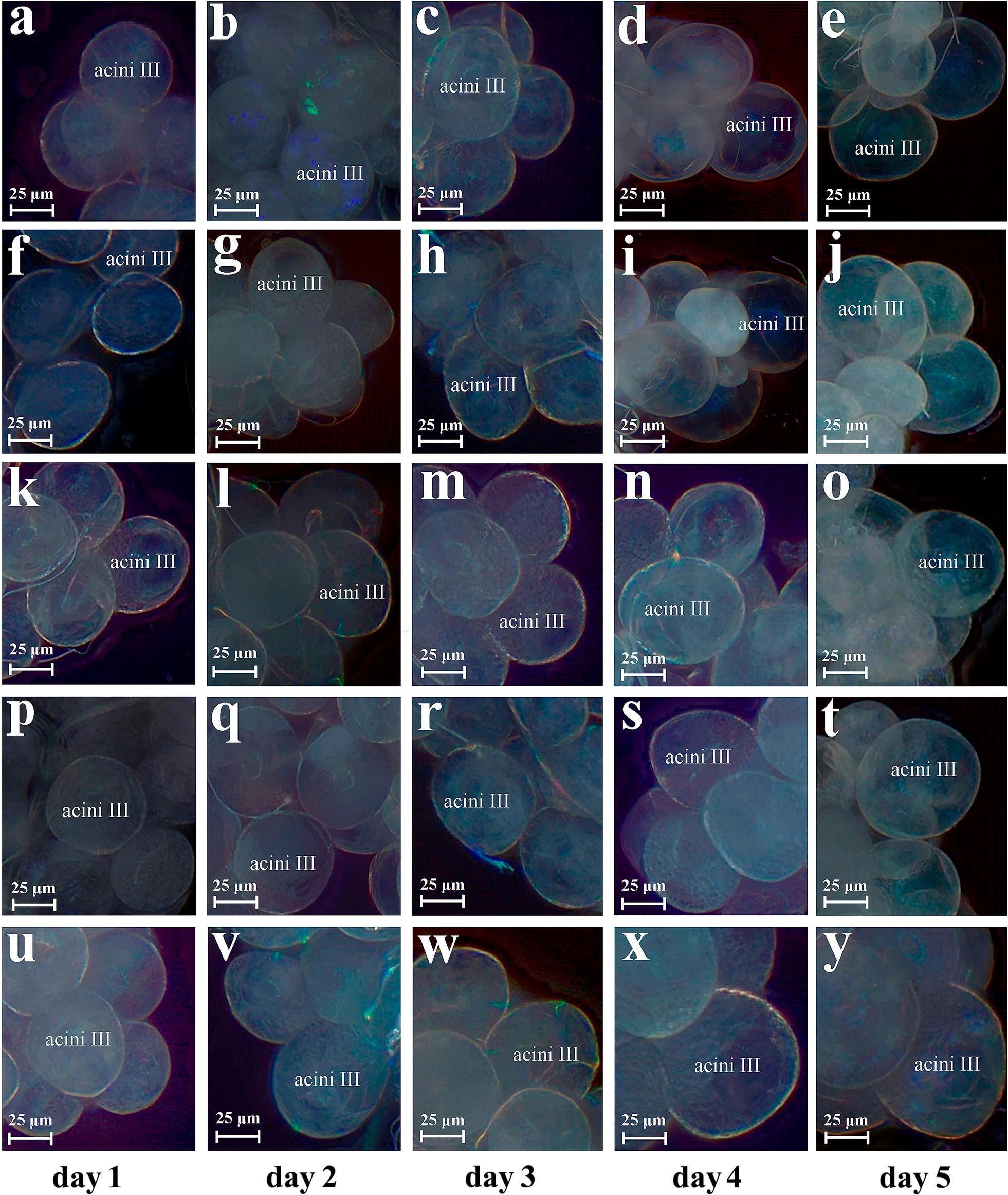
Digital micrographs of salivary gland acinar morphological changes in unfed female *H. longicornis* after RNAi. The time at which the tick bit the host and began sucking blood was recorded as day 0. **a**–**e** Dynein dsRNA injection. **f**–**j** Kinesin dsRNA injection. **k**–**o** Isocitrate dehydrogenase dsRNA injection. **p**–**t** Citrate synthase dsRNA injection. **u**–**y** GFP dsRNA injection, control. *Scale-bars*: 25 µm

After bioinformatics analysis, we found that the protein expression levels of dynein, kinesin, isocitrate dehydrogenase, and citrate synthase were upregulated. In addition, we analyzed the mRNA of these four proteins at the transcriptional level during the four feeding stages of salivary gland development by RT-qPCR (Fig. 5, Additional file 13: Table S11). It was found that the expression of transcription level and protein level was slightly different, but the expression change trend was the same. These proteins may directly affect the physiological function of salivary glands, so we further analysis their function using RNAi. We found that ticks could not suck blood normally after RNAi of dynein, kinesin, isocitrate dehydrogenase, and citrate synthase, and the body length of females represented 45.7 ± 6.1%, 51.2 ± 7.3%, 55.5 ± 8.0% and 53.5 ± 8.6% (mean ± SD) of the body length of females in the control group after blood-feeding for 4 days, respectively. There were significant differences in body length of females after RNAi of dynein, kinesin, isocitrate dehydrogenase and citrate synthase mRNA (Studentʼs t-test: *t*_(2)_ = 10.34, *P* = 0.0092; *t*_(2)_ = 7.45, *P* = 0.0175; *t*_(2)_ = 5.16, *P* = 0.0354; and *t*_(2)_ = 6.23, *P* = 0.0247, respectively) (Fig. 6). Female ticks had a higher mortality rate after RNAi of dynein (47.5%) and kinesin (42.5%). In addition, the development of the salivary glands was greatly affected after RNAi injection. Figure 7 shows the morphological changes of tick salivary glands following injection with different dsRNA. The development of the salivary gland acini in ticks injected with RNA targeting the four genes clearly did not progress to the same extent as that in ticks injected with control GFP dsRNA. The size of acini in the RNAi groups was significantly smaller than that in the control group. After dynein, kinesin, isocitrate dehydrogenase, and citrate synthase mRNA were interfered, the size of type III acini of females were about 60.7 ± 2.7%, 66.7 ± 3.5%, 71.1 ± 3.6% and 80.0 ± 2.1% (mean ± SD) of the size observed in the control group after blood-feeding for 5 days, respectively. There were statistically significant differences in the size of type III acini after RNAi of dynein, kinesin, isocitrate dehydrogenase and citrate synthase mRNA (Studentʼs t-test: *t*_(2)_ = 34.00, *P* = 0.0008; *t*_(2)_ = 26.11, *P* = 0.0014; *t*_(2)_ = 20.19, *P* = 0.0024; and *t*_(2)_ = 8.39, *P* = 0.0139, respectively). In addition, in the RNAi groups, the weights of the engorged female ticks and the egg hatching rates decreased compared with those of the control group (Table 1). Table 2 shows the qPCR results confirming that RNAi knocked down the mRNA of these four genes. The gene expression silencing rate was compared between female ticks RNAi with test genes dsRNA and GFP control dsRNA. The Cq values of the qPCR are provided in Additional file 14: Table S12 and Additional file 15: Table S13. After the injection of dsRNA from the target mRNA, the silencing rates of dynein, kinesin, isocitrate dehydrogenase, and citrate synthase in the salivary glands of female ticks reached 78.3%, 83.3%, 73.2% and 83.1%, respectively. Meanwhile, the silencing rates in the whole ticks were 81.2%, 85.6%, 69.1% and 86.7%, respectively.

**Table 1:** Physiological data for ticks after RNAi

	Mean time required for engorgement (days)^a^	Mortality rate (%)	Mean engorged weight (mg)^a^	Mean no. of eggs^a^	Mean egg hatching rate (%)^a^
Dynein	6.6 ± 0.8	47.5	79.9 ± 19.3^b^	318 ± 46^b^	31.8 ± 8.2^b^
Kinesin	6.5 ± 1.0	42.5	98.8 ± 27.4^b^	453 ± 49^b^	37.2 ± 7.3^b^
Isocitrate dehydrogenase	5.9 ± 0.6	32.5	125.7 ± 25.7^b^	927 ± 63^b^	48.3 ± 5.8^b^
Citrate synthase	5.8 ± 0.8	22.5	112.5 ± 20.1^b^	831 ± 52^b^	51.3 ± 9.1^b^
GFP	5.2 ± 0.6	12.5	213.8 ± 28.2	1801 ± 31	84.7 ± 6.5
No injections	5.1 ± 0.7	15.0	218.9 ± 23.3	1886 ± 45	85.1 ± 4.1

^a^Mean ± standard deviation (SD)

^b^Tick engorged weight, number of eggs, and egg hatching rate was compared between female RNAi with test genes dsRNA and GFP control dsRNA by Studentʼs t-test (*P*-value < 0.05)

**Table 2:** Gene expression silencing by RNAi

Gene	Mean gene expression silencing rate (%)	
	Salivary gland^a^	Whole tick^a^
Dynein	78.3 ± 6.2	81.2 ± 7.7
Kinesin	83.3 ± 7.4	85.6 ± 6.5
Isocitrate dehydrogenase	73.2 ± 11.8	69.1 ± 8.3
Citrate synthase	83.1 ± 10.4	86.7 ± 7.3

^a^Mean ± standard deviation (SD)

*Note*: Gene expression silencing rate was compared between female RNAi with test genes dsRNA and GFP control dsRNA

## Discussion

In the present study, we developed a quantitative iTRAQ proteomics strategy to investigate differential protein expression in salivary glands and thus to further investigate both the development and the functional adjustment of salivary glands in the different feeding stages of female *H. longicornis*. Protein regulation during changes in salivary gland function in female ticks was systematically analyzed. The results of the present study showed that 615 salivary gland proteins were upregulated in partially fed females compared with unfed females (Additional file 4: Table S4).

Tick saliva contains a variety of functional proteins and peptides, such as vasodilators, anticoagulants, inhibitors of platelet aggregation, and immunomodulators that are essential for successful blood-feeding [36]. In addition, it was found that proteins in the saliva of ticks also included heme/iron metabolism-related proteins, oxidation/detoxification proteins, a variety of proteinase inhibitors, and so on [37]. To complete a blood meal at the feeding site ticks secrete proteins produced in the salivary glands that inhibit the host immune response [38, 39]. In the present study, the expression of a protein related to immunosuppressant protein (Da-p36), which can suppress the concanavalin A-induced *in vitro* proliferation of normal murine T-lymphocytes [40], was higher in the salivary glands of semi-engorged and engorged ticks than in those of unfed ticks. Although the change in this protein was not very stable among the three experiments, the data indicated that the expression of this protein was upregulated in the salivary glands of semi-engorged ticks compared with those of unfed ticks. Furthermore, the angiotensin-converting enzyme-like protein found in ticks may have functions similar to those of angiotensin-converting enzyme in mammals [41], which can control blood pressure by regulating fluid volume [42]. This control would allow the tick to continuously feed on host blood.

We further found that two sequences annotated as Kunitz-type serine protease inhibitors were upregulated when ticks sucked blood [43]. However, the homology of these two sequences with those of Kunitz-type serine protease inhibitors was relatively low. Therefore, we cannot reach any definite conclusions about changes in the expression of proteins with anticoagulant functions in tick salivary glands [44]. We believe that the main reason why some small peptides such as antimicrobial peptides [45, 46] were not detected is that we used the FASP method. Although the FASP method can produce pre-labeled samples that are sufficiently clean for iTRAQ labeling and is thus currently an ideal pre-treatment method for iTRAQ, peptides smaller than 10 kDa (sometimes slightly larger than 10 kDa) are washed away by centrifugation before trypsin digestion. In addition, the sequences of functional peptides are very short, which ultimately causes the number of digested peptides that can be identified by MS to be less than that of peptides from digested proteins. Therefore, we did not obtain quantitative information about functional peptides.

The salivary glands of unfed female ticks are undeveloped and protein expression is low [47]. When ticks were exposed to the host but not allowed to feed, some proteins are expressed when female ticks are stimulated to start feeding [9–11]. Once a tick starts sucking blood, salivary gland begins to develop. In the present study, we found that during constant tick blood-feeding the expression of large amounts of proteins increase, with the expression of thrombospondin upregulated in partially fed female salivary glands compared with unfed female salivary glands. We found that the thrombospondin sequence provided in GenBank (XP_002434432.1) showed the greatest homology with thrombospondin 3. Thrombospondin 1 has been confirmed to have antiangiogenic activity in mammals [48]. Although thrombospondin 3 in mammals has not been found to have the same function as thrombospondin 1 [49], it is possible that thrombospondin 3 in ticks could have the same function as thrombospondin 1. The expression of the angiotensin-converting enzyme-like and thrombospondin 3 proteins did not differ significantly among the partially fed, semi-engorged, and engorged stage, which seems to indicate that these proteins are maintained at constant levels in the salivary glands during these periods.

Coat protein complexes I and II (COPI and COPII) can transport proteins between the rough endoplasmic reticulum and the Golgi apparatus [50], which is an essential process in protein synthesis. These two proteins were both upregulated in partially fed compared with unfed female ticks, indicating an acceleration of protein synthesis and transport in the partially fed ticks (Additional file 4: Table S4). In contrast, there was a significant decrease in the expression of COPI in engorged compared with semi-engorged ticks that was associated with a decrease in protein expression during salivary gland degeneration (Additional file 4: Table S4). In partially fed ticks, salivary gland cell division produces large numbers of new cells. Tubulin, a cytoskeleton component [51], was upregulated at this stage, which reflects the massive proliferation and growth of salivary gland cells.

When the salivary glands develop, cell mitosis and meiosis accelerate, and processes that transport cellular cargo become faster. Therefore, developing salivary gland cells require more microtubule motor proteins to transport proteins and organelles [25]. The activity of dynein and kinesin is powered by ATP hydrolysis [52, 53]; these proteins can move along microtubules and transport cargo through interactions with tubulin [54]. In the present study, we found that the expression of these two motor proteins was indeed upregulated in partially fed females compared with unfed females. However, at other blood-feeding stages, there were no differences in the expression of these two motor proteins, indicating that the expression and transport of total proteins in the other three stages may be relatively stable. Through RNAi, we proved that the reduction of these two proteins had a marked effect on the physiological activity of tick salivary glands.

Because a large number of cells are rapidly proliferating, the energy supply during salivary gland development is particularly important. Our results showed that a series of enzymes associated with ATP production, including various enzymes involved in glycolysis, the TCA cycle, the pentose phosphate pathway, and oxidative phosphorylation in respiratory chains, were clearly upregulated. Additional file 4: Table S4 shows the upregulated enzymes involved in glycolysis [55], such as 3-phosphoglycerate kinase, phosphoglycerate mutase, glucose-6-phosphate isomerase, and enolase. Significantly upregulated enzymes involved in the TCA cycle included pyruvate dehydrogenase, citrate synthase, isocitrate dehydrogenase, succinyl-CoA synthetase, succinate dehydrogenase, fumarase, and malate dehydrogenase. Through RNAi, we proved that a lack of citrate synthase and isocitrate dehydrogenase had a marked effect on the physiological activity of tick salivary glands. Furthermore, the enzyme 6-phosphogluconate dehydrogenase, which is involved in the pentose phosphate pathway, was also upregulated. The upregulation of all of these enzymes in ticks should lead to increased NADPH production for the synthesis of lipids, fatty acids, and nucleotides [56]. In addition, the expression of some proteins involved in the respiratory chain protein complexes in the inner mitochondrial membrane, such as NADH-ubiquinone oxidoreductase, succinate dehydrogenase, cytochrome b-c1 complex subunit 2, and F0F1-type ATP synthase, increased significantly. The results indicate that when ticks enter the blood-feeding stage, the TCA cycle and other energy-metabolism pathways in their salivary gland cells become more active so that the ticks can immediately convert carbohydrates, fats, and proteins from the blood of the host into a large amount of ATP *via* the respiratory chain. Ultimately, most organs in ticks were able to develop rapidly after a short duration of blood-feeding.

The mortality of female ticks with dynein and kinesin (47.5% and 42.5%) RNAi was significantly higher than that of the control group (12.5%). In addition, with the significant decrease of engorged weight of female ticks after RNAi, the egg-laying rate, egg quality and hatching rate of female ticks also decreased significantly. The physiological changes of female ticks after RNAi of isocitrate dehydrogenase and citrate synthase were not as obvious as those after RNAi of dynein and kinesin. It is thought that a decrease in these two enzymes involved in the TCA cycle reduces the ATP provided for tick development, but the ticks can obtain ATP in other ways [57]. The motor proteins dynein and kinesin play a decisive role in the transport of substances [58, 59] in the cell of tick, so their reduced expression after RNAi can affect the physiological activity of the tick even more.

A small number of salivary gland proteins appeared to be differentially expressed between semi-engorged and partially fed ticks. According to statistical analysis, 53 proteins, including 12 hypothetical proteins, were upregulated and 34 proteins, including 8 hypothetical proteins, were downregulated in semi-engorged ticks compared with partially fed ticks. The expression levels and change fold of proteins between these two stages are all small, which indicate the function of salivary glands in these two periods is relatively balanced and that the expression levels of the proteins are relatively stable. The female tick enters a rapid feeding stage after mating [60], and the body weight can be increased 100-fold when they are engorged [61]. Previous studies have shown that the expression level of a large number of proteins in ovaries of female ticks changes after mating [62]. In the present study, we found that the expression levels of a large number of proteins in salivary glands also changed after mating (Additional file 4: Table S4), which suggests that energy production should be increased in this stage. However, there were no significant increases in the expression of enzymes related to glycolysis, the TCA cycle, or the pentose phosphate pathway in the salivary glands at this stage. There was little change in the expression of proteins associated with energy metabolism. We speculate that during this period of time, to achieve rapid blood-feeding, the female tick primarily uses energy for development of the salivary glands and other organs, such as the midgut, and that the energy demand of the salivary glands does not increase as much.

The protein expression levels of dynein, kinesin, isocitrate dehydrogenase, and citrate synthase in the salivary glands of female ticks are upregulated. Quantitative results of RT-qPCR showed that the transcription level of these proteins was consistent with the protein level during the development of salivary glands, but the difference was only slight in fold change, which is a frequent occurrence [63]. Although iTRAQ is a more ideal method for protein quantification, and the data generated by us have good reproducibility, it cannot guarantee the accurate quantification of each protein, which is caused by various reasons. Therefore, it may be necessary to use other more advanced quantitative methods to make up for the shortcomings of iTRAQ in the future.

## Conclusions

In this study, the dynamic changes of salivary gland proteins expression in *H. longicornis* from unfed to engorgement were systematically analyzed. This will provide a deeper understanding of the synergistic patterns of proteins in the whole process of salivary gland development to degeneration. Meanwhile, through RNAi on dynein, kinesin, isocitrate dehydrogenase, and citrate synthase, the study investigated the functions and effects of the four proteins on ticks and their salivary glands. The research results provide new protein targets for the control of ticks and tick-borne diseases. The results also provide new protein targets for controlling ticks which can have profound implications for the prevention of tick-borne diseases.

## Supplementary information

**Additional file 1: Table S1.** Statistics for protein quantification information of 115:114.

**Additional file 2: Table S2**. Statistics for protein quantification information of 116:114.

**Additional file 3: Table S3.** Statistics for protein quantification information of 117:114.

**Additional file 4: Table S4.** Statistics for protein quantification information of identified proteins shared among all three biological replicates.

**Additional file 5: Figure S1.** Overview of the reproducibility of iTRAQ quantitative proteomics. **a-c** Correlation coefficients for the partially fed:unfed abundance ratios among 3 replicates; **d-f** Correlation coefficients for the semi-engorged:unfed abundance ratios among 3 replicates; **g-i** Correlation coefficients for the engorged:unfed abundance ratios among 3 replicates.

**Additional file 6: Table S5.** Raw data obtained by cluster analysis using the GProX platform.

**Additional file 7: Figure S2.** GO functional annotations for the differentially expressed proteins in Clusters 1–5. BP: biological process; CC: cellular component; MF: molecular function.

**Additional file 8: Table S6.** Raw data for the KEGG pathway enrichment analysis of differentially expressed proteins in Cluster 1.

**Additional file 9: Table S7.** Raw data for the KEGG pathway enrichment analysis of differentially expressed proteins in Cluster 2.

**Additional file 10: Table S8.** Raw data for the KEGG pathway enrichment analysis of differentially expressed proteins in Cluster 3.

**Additional file 11: Table S9.** Raw data for the KEGG pathway enrichment analysis of differentially expressed proteins in Cluster 4.

**Additional file 12: Table S10.** Raw data for the KEGG pathway enrichment analysis of differentially expressed proteins in Cluster 5

**Additional file 13: Table S11.** Cq values of the RT-qPCR for 4 mRNA in 4 different stages.

**Additional file 14: Table S12.** Cq values of the RT-qPCR in the salivary glands of female ticks.

**Additional file 15: Table S13.** Cq values of the RT-qPCR in the whole female ticks.

## Data Availability

Data supporting the conclusions of this article are included within the article. The proteomic dataset has been submitted to ProteomeXchange *via* the PRIDE database (accession number: PXD013678).

## Abbreviations

iTRAQ: isobaric tags for relative and absolute quantification
FASP: filter-aided sample preparation
TCA: tricarboxylic acid
HCD: high-energy collision-induced dissociation
FDR: false discovery rate
NSAF: normalized spectrum abundance factor
GO: Gene Ontology
KEGG: Kyoto Encyclopedia of Genes and Genomes
GFP: green fluorescent protein
CV: coefficients of variation
COPI and COPII: Coat protein complexes I and II
MS: mass spectrometry
