# Supplementary figures and images for: Salivary gland proteome analysis of developing adult female Haemaphysalis longicornis ticks: molecular motor and TCA cycle-related proteins play an important role throughout development
Source: Parasit Vectors. 2019 Dec 30;12:613. doi: 10.1186/s13071-019-3864-2 (PMC6937756; doi:10.1186/s13071-019-3864-2)

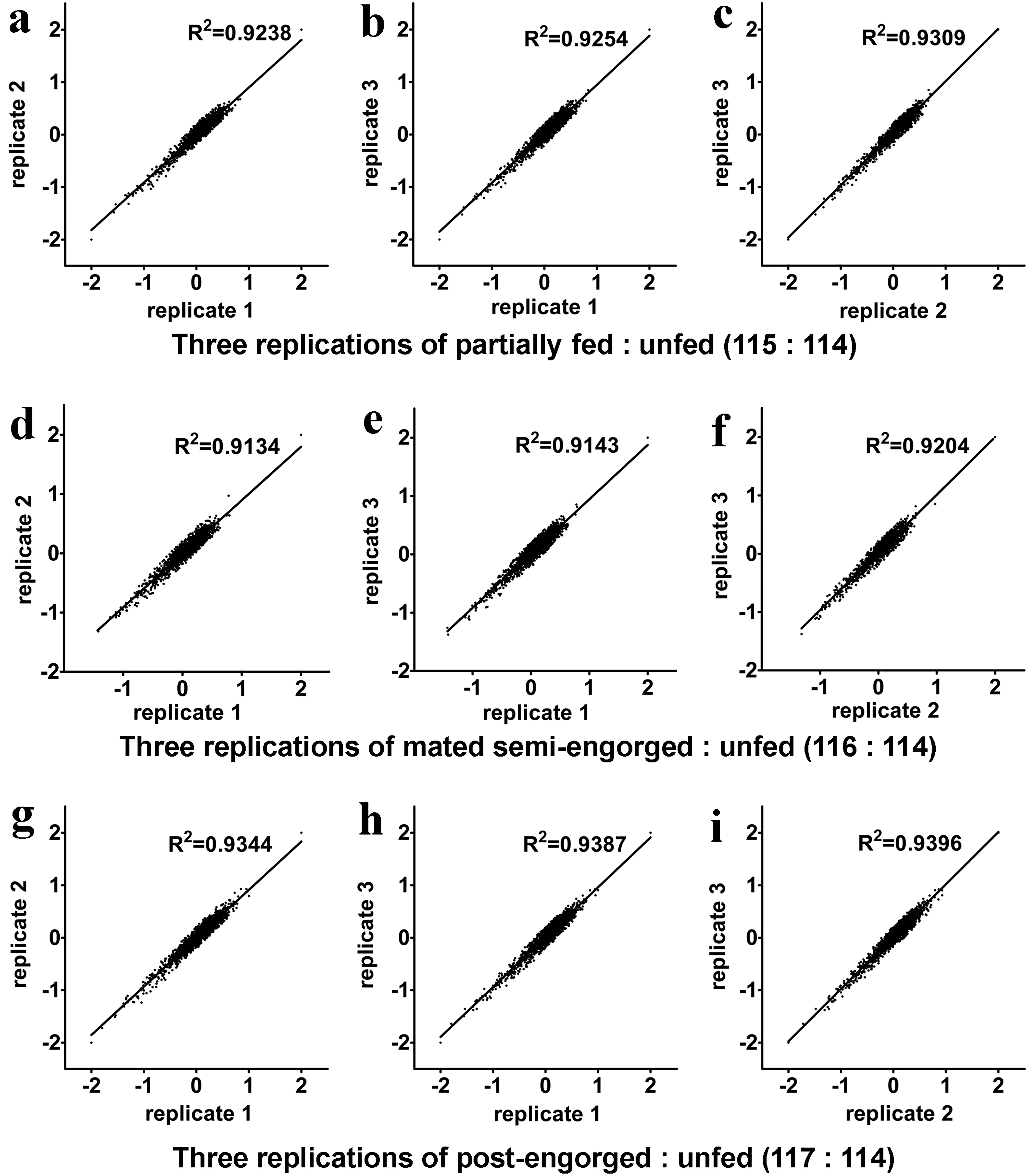

Supplement: Supplementary file 5 — Additional file 5: Figure S1. Overview of the reproducibility of iTRAQ quantitative proteomics. a-c Correlation coefficients for the partially fed:unfed abundance ratios among 3 replicates; d-f Correlation coefficients for the semi-engorged:unfed abundance ratios among 3 replicates; g-i Correlation coefficients for the engorged:unfed abundance ratios among 3 replicates. [file 13071_2019_3864_MOESM5_ESM.tif]
